# 3-(2-Formyl­phen­oxy)propanoic acid

**DOI:** 10.1107/S1600536810038079

**Published:** 2010-09-30

**Authors:** Alain Collas, Christophe M. L. Vande Velde, Frank Blockhuys

**Affiliations:** a Department of Chemistry, University of Antwerp, Universiteitsplein 1, B-2610 Wilrijk, Belgium

## Abstract

In the structure of the title compound, C_10_H_10_O_4_, the carboxyl group forms a catemer motif in the [100] direction instead of the expected dimeric structures. The carboxylic acid group is found in the *syn* conformation and the three-dimensional organization in the crystal is based on C—H⋯O and O—H⋯O interactions.

## Related literature

For the synthesis, see: Zawadowska (1963 ▶); Jarvest *et al.* (2005 ▶). For related structures, see: Gresham *et al.* (1949 ▶); Leiserowitz (1976 ▶); Borthwick (1980 ▶); Kennard *et al.* (1982 ▶); Shockravi *et al.* (2004 ▶); Gao & Ng (2006 ▶). For applications of poly(*p*-phenyl­ene vinyl­ene) oligomers (PPVs), see: Chemla (1987 ▶); Bandyopadhyay & Pal (2003 ▶). For hydrogen bonding and crystal engineering, see: Desiraju (1997 ▶); Steiner (2002 ▶).
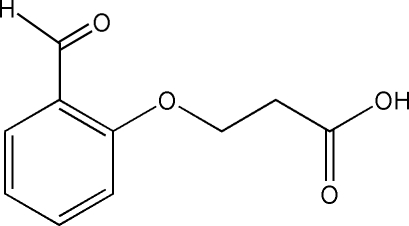


## Experimental

### 

#### Crystal data

C_10_H_10_O_4_
*M* *_r_* = 194.18
Orthorhombic, 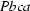

*a* = 15.269 (4) Å
*b* = 7.167 (2) Å
*c* = 17.136 (5) Å
*V* = 1875.2 (9) Å^3^
*Z* = 8
Mo *K*α radiation
μ = 0.11 mm^−1^
*T* = 293 K
0.42 × 0.21 × 0.15 mm

#### Data collection

Enraf–Nonius CAD-4 diffractometer
1718 measured reflections
1718 independent reflections
964 reflections with *I* > 2σ(*I*)
3 standard reflections every 60 min  intensity decay: 1%

#### Refinement

*R*[*F* ^2^ > 2σ(*F* ^2^)] = 0.047
*wR*(*F* ^2^) = 0.119
*S* = 1.02
1718 reflections
167 parameters
All H-atom parameters refined
Δρ_max_ = 0.17 e Å^−3^
Δρ_min_ = −0.21 e Å^−3^

### 

Data collection: *CAD-4 EXPRESS* (Enraf–Nonius, 1994 ▶); cell refinement: *CAD-4 EXPRESS*; data reduction: *XCAD4* (Harms & Wocadlo, 1995 ▶); program(s) used to solve structure: *SHELXS97* (Sheldrick, 2008 ▶); program(s) used to refine structure: *SHELXL97* (Sheldrick, 2008 ▶); molecular graphics: *ORTEP-3* (Farrugia, 1997 ▶) and *PLATON* (Spek, 2009 ▶); software used to prepare material for publication: *WinGX* (Farrugia, 1999 ▶).

## Supplementary Material

Crystal structure: contains datablocks I, global. DOI: 10.1107/S1600536810038079/zl2308sup1.cif

Structure factors: contains datablocks I. DOI: 10.1107/S1600536810038079/zl2308Isup2.hkl

Additional supplementary materials:  crystallographic information; 3D view; checkCIF report

## Figures and Tables

**Table 1:** Hydrogen-bond geometry (Å, °)

*D*—H⋯*A*	*D*—H	H⋯*A*	*D*⋯*A*	*D*—H⋯*A*
O3—H41⋯O4^i^	0.94 (4)	1.72 (4)	2.618 (3)	158 (3)
C21—H21*a*⋯O1^ii^	1.00 (3)	2.64 (3)	3.409 (4)	134.5 (2)
C22—H22*b*⋯O1^iii^	0.96 (3)	2.46 (3)	3.187 (4)	132 (2)
C21—H21*a*⋯O3^iv^	1.00 (3)	2.60 (2)	3.456 (3)	144 (2)
C3—H3⋯O4^v^	0.96 (2)	2.71 (2)	3.536 (4)	144.9 (2)

Symmetry codes: (i) 
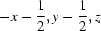
; (ii) 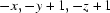; (iii) 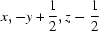; (iv) 
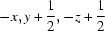
; (v) 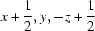.

**Fig. 1.**
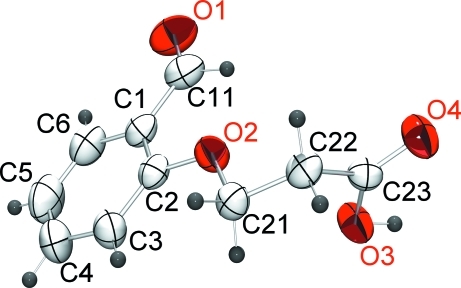
Molecular structure of the title compound with the numbering scheme. Displacement ellipsoids are drawn at the 50% probability level and hydrogen atoms are represented as spheres with an arbitrary radius.

**Fig. 2.**
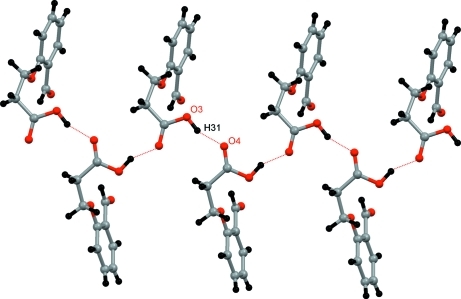
Representation of the infinite chain of hydrogen bonds involving the carboxyl moiety through the crystal structure of the title compound. See Table 1 for details.

**Fig. 3.**
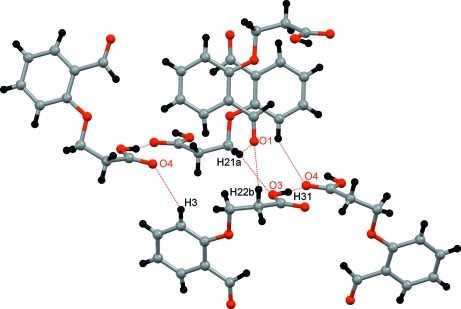
Additional short contacts in the crystal structure of the title compound. See Table 1 for details.

## supplementary crystallographic information

### Comment

The title compound was synthesized as a precursor for asymmetric PPV-type [poly(*p*-phenylene vinylene)] oligomers. These compounds are promising candidates as the active materials in organic memories (Bandyopadhyay & Pal, 2003) and as non-linear optical (NLO) materials with a high first-order hyperpolarizability (Chemla, 1987). Besides the condition that these oligomers should bear donor and acceptor substituents connected by a π-system, it is of critical importance for their usefulness as an NLO material and as a bistable organic memory material that the crystal packing is non-centrosymmetric. In particular, the compound should crystallize in a polar space group. To influence the crystal packing by means of the crystal engineering methodology in order to meet this criterion, it is necessary to introduce certain statistically well chosen synthons (Desiraju, 1997). Therefore, we opted for carboxylic acid functional groups (powerful hydrogen bond donors) in the basic structure of the organic semiconductor. To combine the electronic (A-π-D) and the structural (non-centrosymmetric space group) requirements, we used the Williamson ether synthesis to prepare the title compound as a building block for a PPV-based semiconductor bearing a carboxylic acid moiety.

The geometry of the aldehyde contains no surprises. The methylene fragments are in synclinal conformation and the torsion angle is 65.9 (3)°. As a result and due to the repulsion between the lone pairs of oxygen atoms O2 and O3, the carboxylic acid group, found in the *syn* conformation, is twisted out of the plane of the phenyl ring. The O2···O3 distance is 3.034 (3) Å and this is the only contact of the ether oxygen atom. The distances in the carboxylic acid moiety are in accordance with the expected values for non-disordered carboxylic acids (Leiserowitz, 1976): the carbonyl (C=O) bond length C23—O4 is 1.209 (3) Å and the C—O single bond length C23—O3 is 1.306 (3) Å. The angles of the carboxyl moiety contain no surprises (Borthwick, 1980): C23—O3—H31 is 110 (2)° and O4—C23—O3 is 121.9 (2)°.

Two intramolecular CH···O hydrogen bonds involving the aldehyde group can be identified: the relevant parameters of C6—H6···O1 and C11—H1···O2 are given in Table 1, entries 1 and 2. The incorporation of a carboxylic acid group was expected to yield the corresponding dimer synthon, but the crystal structure of the title compound reveals a catemer motif in the [1 0 0] direction (Fig. 2), consisting of an infinite chain of hydrogen bonds. The O3···O4 distance is 2.618 (3) Å (Table 1, entry 3) which is not surprising for a hydrogen bond of moderate strength (Steiner, 2002). The chains are intertwined through O1···*Cg* contacts in which the aldehyde oxygen atom contacts the center of the aromatic ring [O1···*Cg*^i^ 3.508 (3) Å, C11—O1···*Cg*^i^ 78.47 (17)°, symm. code i = 1-*x*, -*y*, -*z*]. O1 is also involved in weak CH···O hydrogen bonds with H21*a* (Table 1, entry 4) and H22*b* (Table 1, entry 5); the latter links the infinite hydrogen bonded chains in the [0 0 1] direction (Fig. 3). Two additional hydrogen bonds can be observed: H21*a* contacts O3 and H3 contacts O4 (Table 1, entries 6 and 7, respectively).

### Experimental

Salicylic aldehyde and 3-chloropropanoic acid were obtained from ACROS and used as received. The general procedure of Gresham was followed (Gresham *et al.*, 1949). 8 g of NaOH in 20 ml of distilled water were added to a stirred solution of 12.2 g (0.1 mol) of salicylic aldehyde and 10.9 g (0.1 mol) of 3-chloropropanoic acid in 80 ml of distilled water. After heating under reflux for 4 h, the mixture was acidified with 19.5 ml of conc. HCl. Unreacted salicylic aldehyde was removed by steam distillation and the resulting mixture was placed in the refrigerator. The resulting tan-coloured needles were collected by filtration in a 20% yield. M.p. 381 K (uncorrected). Crystals suitable for the diffraction experiment were grown by slow evaporation of a 50:50 chloroform:toluene solvent mixture, analogous with the experiments of Kennard (Kennard *et al.*, 1981). ^1^H NMR (CD_3_OD, 400 MHz, TMS): δ 2.84 (t, 2*H*, 6.00 Hz, H22), 4.39 (t, 2H, 6.00 Hz, H21), 7.05 (td, 1H, 7.44 and 0.88 Hz, H5), 7.18 (d, 1H, 8.10 Hz, H3), 7.61 (td, 1H, 7.32 and 1.88 Hz, H4), 7.75 (dd, 1H, 7.72 and 1.80 Hz, H6), 10.39 (d, 1H, 0.80 Hz, H21*b*); the proton of the carboxylic acid can not be seen. ^13^C NMR (CDCl_3_, 100 MHz, TMS): δ 34.17 (C22), 63.83 (C21), 112.66 (C3), 121.33 (C5), 125.08 (C1), 128.61 (C6), 135.99 (C4), 160.75 (C2), 175.97 (C11), 189.79 (C23).

### Refinement

The positions of the hydrogen atoms were derived from the electron density difference map and *X*—H bonding distances are situated between 0.89 (3) Å and 1.03 (3) Å.

### Crystal data

**Table:**

C_10_H_10_O_4_	*D*_x_ = 1.376 Mg m^−^^3^
*M**_r_* = 194.18	Melting point: 381 K
Orthorhombic, *P**b**c**a*	Mo *K*α radiation, λ = 0.71073 Å
Hall symbol: -P 2ac 2ab	Cell parameters from 25 reflections
*a* = 15.269 (4) Å	θ = 6.2–19.0°
*b* = 7.167 (2) Å	µ = 0.11 mm^−^^1^
*c* = 17.136 (5) Å	*T* = 293 K
*V* = 1875.2 (9) Å^3^	Block, colourless
*Z* = 8	0.42 × 0.21 × 0.15 mm
*F*(000) = 816	

### Data collection

**Table:**

Enraf–Nonius CAD-4 diffractometer	*R*_int_ = 0.000
Radiation source: fine-focus sealed tube	θ_max_ = 25.3°, θ_min_ = 2.4°
graphite	*h* = −18→0
ω/2θ scans	*k* = −8→0
1718 measured reflections	*l* = 0→20
1718 independent reflections	3 standard reflections every 60 min
964 reflections with *I* > 2σ(*I*)	intensity decay: 1%

### Refinement

**Table:**

Refinement on *F*^2^	Primary atom site location: structure-invariant direct methods
Least-squares matrix: full	Secondary atom site location: difference Fourier map
*R*[*F*^2^ > 2σ(*F*^2^)] = 0.047	Hydrogen site location: inferred from neighbouring sites
*wR*(*F*^2^) = 0.119	All H-atom parameters refined
*S* = 1.02	*w* = 1/[σ^2^(*F*_o_^2^) + (0.0516*P*)^2^] where *P* = (*F*_o_^2^ + 2*F*_c_^2^)/3
1718 reflections	(Δ/σ)_max_ < 0.001
167 parameters	Δρ_max_ = 0.17 e Å^−^^3^
0 restraints	Δρ_min_ = −0.21 e Å^−^^3^

### Special details

**Table:**

Geometry. All e.s.d.'s (except the e.s.d. in the dihedral angle between two l.s. planes) are estimated using the full covariance matrix. The cell e.s.d.'s are taken into account individually in the estimation of e.s.d.'s in distances, angles and torsion angles; correlations between e.s.d.'s in cell parameters are only used when they are defined by crystal symmetry. An approximate (isotropic) treatment of cell e.s.d.'s is used for estimating e.s.d.'s involving l.s. planes.
Refinement. Refinement of *F*^2^ against ALL reflections. The weighted *R*-factor *wR* and goodness of fit *S* are based on *F*^2^, conventional *R*-factors *R* are based on *F*, with *F* set to zero for negative *F*^2^. The threshold expression of *F*^2^ > σ(*F*^2^) is used only for calculating *R*-factors(gt) *etc*. and is not relevant to the choice of reflections for refinement. *R*-factors based on *F*^2^ are statistically about twice as large as those based on *F*, and *R*- factors based on ALL data will be even larger.

### Fractional atomic coordinates and isotropic or equivalent isotropic displacement parameters (Å^2^)

**Table:**

	*x*	*y*	*z*	*U*_iso_*/*U*_eq_	
H21a	0.0220 (14)	0.495 (4)	0.3039 (14)	0.049 (7)*	
H22b	−0.1019 (16)	0.478 (4)	0.2296 (16)	0.063 (9)*	
H22a	−0.1276 (16)	0.540 (4)	0.3140 (14)	0.058 (9)*	
H21b	0.0046 (17)	0.281 (4)	0.2824 (15)	0.058 (8)*	
H5	0.2323 (19)	0.109 (4)	0.5447 (16)	0.073 (10)*	
H3	0.1355 (15)	0.315 (4)	0.3435 (14)	0.049 (8)*	
H41	−0.1930 (19)	0.040 (6)	0.2804 (19)	0.104 (13)*	
H4	0.2503 (19)	0.196 (4)	0.4169 (15)	0.058 (9)*	
H6	0.092 (2)	0.133 (4)	0.6011 (18)	0.081 (11)*	
H1	−0.100 (2)	0.276 (4)	0.5065 (19)	0.099 (12)*	
O3	−0.14623 (11)	0.1255 (3)	0.28392 (12)	0.0509 (6)	
O2	−0.02664 (10)	0.3422 (3)	0.39246 (10)	0.0464 (5)	
O4	−0.25446 (11)	0.3279 (2)	0.28021 (11)	0.0562 (6)	
C23	−0.17679 (16)	0.2958 (3)	0.28326 (14)	0.0384 (6)	
C2	0.04663 (17)	0.2827 (3)	0.43064 (16)	0.0414 (7)	
C1	0.03508 (17)	0.2307 (4)	0.50805 (16)	0.0443 (7)	
C21	−0.01910 (17)	0.3884 (4)	0.31172 (16)	0.0426 (7)	
O1	−0.06445 (15)	0.2082 (3)	0.61361 (12)	0.0779 (7)	
C22	−0.10849 (17)	0.4449 (4)	0.28378 (19)	0.0421 (7)	
C3	0.12868 (17)	0.2735 (4)	0.39650 (18)	0.0511 (8)	
C6	0.1071 (2)	0.1672 (5)	0.5502 (2)	0.0625 (9)	
C11	−0.0514 (2)	0.2416 (4)	0.54553 (18)	0.0565 (8)	
C4	0.1983 (2)	0.2109 (5)	0.4398 (2)	0.0690 (10)	
C5	0.1876 (2)	0.1563 (6)	0.5161 (2)	0.0761 (11)	

### Atomic displacement parameters (Å^2^)

**Table:**

	*U*^11^	*U*^22^	*U*^33^	*U*^12^	*U*^13^	*U*^23^
O3	0.0400 (10)	0.0306 (11)	0.0823 (15)	0.0022 (8)	0.0009 (11)	0.0024 (10)
O2	0.0428 (10)	0.0563 (12)	0.0401 (11)	0.0032 (9)	0.0023 (9)	0.0062 (10)
O4	0.0409 (10)	0.0359 (10)	0.0919 (15)	0.0035 (9)	−0.0046 (11)	−0.0016 (11)
C23	0.0428 (15)	0.0324 (14)	0.0401 (15)	0.0047 (12)	0.0018 (13)	0.0013 (12)
C2	0.0481 (16)	0.0314 (14)	0.0449 (17)	−0.0040 (12)	−0.0047 (13)	−0.0030 (12)
C1	0.0533 (18)	0.0353 (15)	0.0442 (17)	−0.0071 (13)	−0.0018 (13)	−0.0035 (13)
C21	0.0438 (16)	0.0416 (17)	0.0424 (16)	−0.0065 (14)	0.0018 (13)	0.0020 (14)
O1	0.1120 (18)	0.0752 (16)	0.0466 (14)	−0.0116 (14)	0.0176 (12)	0.0007 (12)
C22	0.0500 (16)	0.0312 (16)	0.0451 (18)	−0.0034 (12)	−0.0004 (15)	0.0009 (14)
C3	0.0437 (16)	0.0558 (18)	0.0537 (19)	−0.0044 (14)	−0.0007 (16)	0.0009 (16)
C6	0.075 (2)	0.059 (2)	0.054 (2)	−0.0036 (18)	−0.011 (2)	−0.0018 (17)
C11	0.077 (2)	0.0448 (19)	0.047 (2)	−0.0066 (17)	0.0067 (18)	−0.0035 (15)
C4	0.0453 (19)	0.084 (3)	0.077 (3)	0.0022 (18)	−0.0088 (19)	−0.009 (2)
C5	0.072 (3)	0.080 (3)	0.077 (3)	0.009 (2)	−0.031 (2)	0.000 (2)

### Geometric parameters (Å, °)

**Table:**

O3—C23	1.306 (3)	C21—H21b	0.99 (3)
O3—H41	0.95 (4)	O1—C11	1.208 (3)
O2—C2	1.364 (3)	C22—H22b	0.96 (3)
O2—C21	1.427 (3)	C22—H22a	0.90 (3)
O4—C23	1.209 (3)	C3—C4	1.371 (4)
C23—C22	1.493 (3)	C3—H3	0.96 (2)
C2—C3	1.384 (4)	C6—C5	1.363 (5)
C2—C1	1.389 (4)	C6—H6	0.93 (3)
C1—C6	1.392 (4)	C11—H1	1.03 (3)
C1—C11	1.471 (4)	C4—C5	1.375 (5)
C21—C22	1.502 (4)	C4—H4	0.89 (3)
C21—H21a	1.00 (3)	C5—H5	0.91 (3)
			
C23—O3—H41	110 (2)	C21—C22—H22b	106.2 (15)
C2—O2—C21	118.1 (2)	C23—C22—H22a	108.5 (17)
O4—C23—O3	121.9 (2)	C21—C22—H22a	108.3 (17)
O4—C23—C22	123.3 (2)	H22b—C22—H22a	113 (2)
O3—C23—C22	114.8 (2)	C4—C3—C2	119.2 (3)
O2—C2—C3	123.7 (3)	C4—C3—H3	121.8 (14)
O2—C2—C1	116.0 (2)	C2—C3—H3	118.9 (15)
C3—C2—C1	120.4 (3)	C5—C6—C1	120.6 (4)
C6—C1—C2	118.9 (3)	C5—C6—H6	127 (2)
C6—C1—C11	120.0 (3)	C1—C6—H6	112.3 (19)
C2—C1—C11	121.1 (3)	O1—C11—C1	124.0 (3)
O2—C21—C22	107.3 (2)	O1—C11—H1	123.9 (19)
O2—C21—H21a	111.0 (14)	C1—C11—H1	112.0 (19)
C22—C21—H21a	108.9 (14)	C3—C4—C5	121.0 (3)
O2—C21—H21b	110.0 (16)	C3—C4—H4	119.5 (18)
C22—C21—H21b	112.4 (15)	C5—C4—H4	119.2 (18)
H21a—C21—H21b	107 (2)	C6—C5—C4	119.9 (4)
C23—C22—C21	116.3 (2)	C6—C5—H5	118.0 (19)
C23—C22—H22b	104.2 (16)	C4—C5—H5	122.1 (19)
			
C21—O2—C2—C3	3.4 (4)	O2—C2—C3—C4	−179.7 (3)
C21—O2—C2—C1	−176.9 (2)	C1—C2—C3—C4	0.6 (4)
O2—C2—C1—C6	179.5 (2)	C2—C1—C6—C5	0.0 (4)
C3—C2—C1—C6	−0.8 (4)	C11—C1—C6—C5	179.9 (3)
O2—C2—C1—C11	−0.4 (4)	C6—C1—C11—O1	4.3 (4)
C3—C2—C1—C11	179.3 (3)	C2—C1—C11—O1	−175.8 (3)
C2—O2—C21—C22	178.8 (2)	C2—C3—C4—C5	0.4 (5)
O4—C23—C22—C21	162.1 (3)	C1—C6—C5—C4	1.0 (5)
O3—C23—C22—C21	−20.0 (4)	C3—C4—C5—C6	−1.2 (6)
O2—C21—C22—C23	−65.9 (3)		

### Hydrogen-bond geometry (Å, °)

**Table:**

*D*—H···*A*	*D*—H	H···*A*	*D*···*A*	*D*—H···*A*
C6—H6···O1	0.94 (3)	2.46 (3)	2.851 (4)	105 (2)
C11—H1···O2	1.03 (3)	2.30 (3)	2.746 (4)	105 (2)
O3—H41···O4^i^	0.94 (4)	1.72 (4)	2.618 (3)	158 (3)
C21—H21a···O1^ii^	1.00 (3)	2.64 (3)	3.409 (4)	134.5 (2)
C22—H22b···O1^iii^	0.96 (3)	2.46 (3)	3.187 (4)	132 (2)
C21—H21a···O3^iv^	1.00 (3)	2.60 (2)	3.456 (3)	144 (2)
C3—H3···O4^v^	0.96 (2)	2.71 (2)	3.536 (4)	144.9 (2)

Symmetry codes: (i) −*x*−1/2, *y*−1/2, *z*; (ii) −*x*, −*y*+1, −*z*+1; (iii) *x*, −*y*+1/2, *z*−1/2; (iv) −*x*, *y*+1/2, −*z*+1/2; (v) *x*+1/2, *y*, −*z*+1/2.
